# *PDGFRA* K385 mutants in myxoid glioneuronal tumors promote receptor dimerization and oncogenic signaling

**DOI:** 10.1038/s41598-024-57859-5

**Published:** 2024-03-26

**Authors:** Laurence de Villenfagne, Ariane Sablon, Jean-Baptiste Demoulin

**Affiliations:** De Duve Institute, University of Louvain, Avenue Hippocrate 75, Box B1.74.05, 1200 Brussels, Belgium

**Keywords:** Growth factor signalling, CNS cancer, Oncogenes, Targeted therapies

## Abstract

Myxoid glioneuronal tumors (MGNT) are low-grade glioneuronal neoplasms composed of oligodendrocyte-like cells in a mucin-rich stroma. These tumors feature a unique dinucleotide change at codon 385 in the platelet-derived growth factor receptor α (encoded by the *PDGFRA* gene), resulting in the substitution of lysine 385 into leucine or isoleucine. The functional consequences of these mutations remain largely unexplored. Here, we demonstrated their oncogenic potential in fibroblast and Ba/F3 transformation assays. We showed that the K385I and K385L mutants activate STAT and AKT signaling in the absence of ligand. Co-immunoprecipitations and BRET experiments suggested that the mutations stabilized the active dimeric conformation of the receptor, pointing to a new mechanism of oncogenic PDGF receptor activation. Furthermore, we evaluated the sensitivity of these mutants to three FDA-approved tyrosine kinase inhibitors: imatinib, dasatinib, and avapritinib, which effectively suppressed the constitutive activity of the mutant receptors. Finally, K385 substitution into another hydrophobic amino acid also activated the receptor. Interestingly, K385M was reported in a few cases of brain tumors but not in MGNT. Our results provide valuable insights into the molecular mechanism underlying the activation of PDGFRα by the K385I/L mutations, highlighting their potential as actionable targets in the treatment of myxoid glioneuronal tumors.

## Introduction

Myxoid glioneuronal tumors (MGNT) constitute a new entity in the WHO classification of central nervous system tumors (CNS). They were previously considered as a subgroup of dysembryoplastic neuroepithelial tumors^1^. MGNT are low-grade neoplasms that preferentially arise in the septum pellucidum or in the periventricular white matter of the lateral ventricles^2–9^. They affect mostly children and young adults, who develop symptoms such as epilepsy, headache or behavioral disturbances. Several studies reported an indolent disease course after gross total resection of the tumor mass. Nevertheless, recurrences do occur and may require chemotherapy and radiotherapy^3^. Long term clinical outcomes and optimal treatment for MGNT remain unknown.

These tumors feature an unusual dinucleotide mutation at codon 385 of platelet-derived growth factor receptor alpha (PDGFRα, encoded by the *PDGFRA* gene), replacing a lysine by a leucine or an isoleucine (K385I/L). PDGFRα is a receptor-tyrosine kinase, the structure of which comprises five extracellular immunoglobulin-like domains, a transmembrane domain, an inhibitory juxtamembrane domain, and a tyrosine kinase domain. PDGFRα binds to platelet-derived growth factor (PDGF) isoforms A, B and C^10,11^. Ligand binding induces the receptor dimerization, activation of the intracellular tyrosine kinase domain, and, ultimately, activation of signaling mediators such as mitogen-activated protein kinases (MAPK), phospholipase C γ (PLCγ), signal transduction and activators of transcription (STAT1, STAT3, STAT5), phosphatidylinositol-3 kinase (PI3K) and AKT. PDGFRα is expressed in mesenchymal cells and oligodendrocyte progenitors, playing an important role in brain development^11^.

*PDGFRA* is also a well-characterized oncogene, which can be targeted by tyrosine kinase inhibitors (TKI), such as imatinib, dasatinib or avapritinib^12^. For instance, chromosomal alterations induce *PDGFRA* fusion with various partner genes, such as *FIP1L1*, in a rare life-threatening hematological malignancy associated with severe hypereosinophilia, which shows an exceptional response rate to imatinib monotherapy^13^. *PDGFRA* is mutated in about 6% of gastrointestinal stromal tumors (GIST). Activating mutations occur in several hotspots in the intracellular juxtamembrane and kinase domains. Some mutant receptors are sensitive to imatinib, while others, such as PDGFRα D842V, show primary resistance to this drug and can only be blocked by more recent TKI, such as avapritinib^14^. Activation of PDGFRα by gene amplification was also reported in 10 to 30% of glioma cases, including glioblastoma multiforme and high-grade pediatric glioma, in which TKI did not offer any benefit^15^.

The K385I/L substitutions associated with MGNT are located in the fourth extracellular immunoglobulin-like domain (D4) of the receptor. The impact of these variants on the receptor activity is unknown. Moreover, the D4 domain structure in PDGFRα has not been solved. Based on receptors of the same family, such as KIT and PDGFRβ, D4 is thought to homodimerize upon ligand binding, undergo a conformational change and contribute to signal transduction^16^. The aim of the present study was to characterize the K385I/L variants. We showed that these mutant receptors were constitutively activated and stimulated cell proliferation, which is consistent with an oncogenic role in MGNT. In addition, the mutations increased the receptor dimerization, which represents a novel mechanism of PDGF receptor activation by a point mutation. Finally, mutant receptors remained sensitive to imatinib, dasatinib and avapritinib.

## Material and methods

### Cell culture, reagents and vectors

The HEK293T and NIH3T3 cell lines were purchased from ATCC (Manassas, VA, USA) and cultured in Dulbecco’s Modified Eagle’s Medium (DMEM, Gibco, Life Technologies, Grand Island, NY, USA) supplemented with 10% fetal bovine serum (FBS, GE Healthcare, Diegem, Belgium) and antibiotics (50 U/ml penicillin and 50 μg/ml streptomycin). The Ba/F3 cell line was cultured in the same medium supplemented with IL-3 (500 U/ml). The U-87 MG cell line, a kind gift from Pr. B. Van den Eynde (Ludwig Cancer research, Brussels, Belgium) was cultured in Iscove’s Modified Dulbecco’s Medium (IMDM, Gibco) supplemented with 10% FBS and antibiotics. Western blots were performed as described with anti-phospho-Y754 PDGFRα (#12911, Santa Cruz Biotechnology, Dallas, Texas, USA), anti-PDGFRα (#5241S, Cell Signaling Technology, Danvers, MA, USA), anti-phospho-S473 AKT (#9271, Cell Signaling Technology), anti-AKT (#9272, Cell Signaling Technology), anti-HA-tag (#3724, Cell Signaling Technology), anti-Myc (#2272, Cell Signaling Technology), and anti-β-actin (#A5441, Sigma-Aldrich, Saint-Louis, MO, USA). Imatinib and dasatinib were purchased from LC Laboratories (Woburn, MA, USA), and avapritinib from Selleckchem (Houston, TX, USA). PDGF-BB was purchased from PeproTech (Rocky Hill, NJ, USA). The *PDGFRA* coding sequence was inserted into pMSCV and pEF-myc-cyto vectors^17,18^. Point mutations were introduced by site-directed mutagenesis according to the QuickChange XL-II kit protocol (Stratagene, La Jolla, CA, USA). The nucleotide sequence of each construct was verified by Sanger sequencing (Eurofins).

### Retroviral infection

Retroviral particles were produced in HEK293T cells. The day after seeding (in T75 flasks), 18 µg of pMSCV-PDGFRA were co-transfected with the packaging plasmid pCMV-Gag-Pol (6 µg), and the envelope plasmid pCMV-VSVg (3 µg) using the calcium phosphate precipitate method as described^19^. After 48 h of transfection, supernatants were harvested, filtered and added on U-87 MG or Ba/F3 cells in the presence of polybrene (8 µg/ml). The next day, cells were washed and selected with puromycin (1 µg/ml) for 5 days.

### Flow cytometry

Ba/F3 cells were harvested, washed in PBS and stained with a primary mouse anti-PDGFRα antibody (#MAB1264, R&D systems, Minneapolis, MN, USA, MAB1264) for 30 min at 4 °C. Cells were then washed and incubated with an anti-mouse secondary antibody conjugated to phycoerythrin (#715-116-150, Jackson Immunoresearch, West Grove, PA, USA) for 30 min at 4 °C in the dark. PDGFRα expression was analyzed using a FACSVerse flow cytometer (BD Biosciences).

### Protein extraction, western blot and immunoprecipitation

Cells were starved overnight in the absence of serum, then stimulated as indicated with PDGF-BB. After washing cells with PBS, proteins were extracted in lysis buffer (25 mM Tris–HCl pH 7.4, 150 mM NaCl, 6 mM EDTA, 10% glycerol, 1% Triton X-100, 1 mM sodium orthovanadate, 1 mM Pefabloc, and 1 µg/ml aprotinin) for 20 min on ice. Lysates were cleared by high-speed centrifugation for 10 min at 4 °C, and protein concentrations were measured using the BCA Protein Assay Kit (Thermo Fisher Scientific, Waltham, MA, USA). Protein samples (30 µg) were mixed with 4× Laemmli sample buffer (0.2 M Tris–HCl pH 6.8, 8% SDS, 0.4% bromophenol blue, 40% glycerol, 2.8% β-mercaptoethanol), separated in Novex Tris–Glycine 4–12% precast gels (Thermo Fisher Scientific) and blotted onto PVDF membranes (GE Healthcare). The membranes were blocked in 5% non-fat dried milk powder and then incubated overnight at 4 °C with the indicated antibodies. Some membranes were cut at the level of the 100 kDa marker to hybridize the two parts with different antibodies. Images were captured using the Fusion Solo S system (Vilber Lourmat, Marne-la-Vallée, France) and were not subsequently modified except for cropping to the size shown in figures. Full blots are available as supplementary material. For co-immunoprecipitation assays, cells were lysed in RIPA buffer (50 mM Tris/HCl pH 7.4, 150 mM NaCl, 1 mM EDTA, 0.25% sodium deoxycholate, 1% NP-40, 1 mM sodium orthovanadate, 1 mM Pefabloc, and 1 µg/ml aprotinin). Protein lysates (800 µg) were incubated overnight with an anti-HA antibody at 4 °C. Immunoprecipitates were then captured by Protein A/G UltraLink Resin (#53133, Thermo Fisher) for 1 h 30 at 4 °C and washed 3 times before being resuspended in 2× Laemmli buffer for western blot analysis.

### Luciferase reporter assay

U-87 cells were seeded in 24-well plates (100 000 cells/well in duplicates). The next day, cells were co-transfected with wild-type (WT) or mutant pEF-myc-cyto-PDGFRA (250 ng), pGRR5-Luc (250 ng) and pEF1-β-Galactosidase (Invitrogen, Carlsbad, CA, USA) (250 ng) as internal control, using TurboFect (2 µL, R0531, ThermoFisher), as recommended by the manufacturer. After 4 h, cells were washed 3 times in PBS and treated with PDGF-BB or imatinib in serum-free medium. After 24 h, cells were washed and lysed in 100 µl of passive lysis buffer (25 mM Tri-Base pH 7.8, 2 mM DTT, 2 mM 1,2-diaminocyclohexane-N,N,N′,N′-tetraacetic acid, 10% glycerol, 1% Triton X-100). Luminescence was measured in cell lysates in presence of Luciferase Reagent (#E1483, Promega, Leiden, The Netherlands) using a GLOMAX instrument (Promega). The luciferase activity was normalized to the β-galactosidase activity, which was measured at 405 nm after adding β-galactosidase substrate solution (1:1) (165 mM Na_2_HPO_4_, 38 mM NaH_2_PO_4_, 2 mM MgCl_2_, 0.5% β-mercaptoethanol, 4.4 mM o-nitrophenyl-β-d-galactopyranoside) as described^19^.

### Nano-bioluminescence resonance energy transfer

PDGFRα coding sequence, truncated after residue R979, was cloned in frame into a modified Nano-Luciferase vector or a HaloTag vector (Promega) to generate C-terminal fusion constructs. HEK293T cells were transiently transfected with respectively 1 ng of these constructs with the TransIT-LT1 Transfection Reagent (Mirus, Madison, WI, USA) in a 96-well plate. After 4 h, the fluorescent NanoBRET HaloTag 618 Ligand was added (#N1662, Promega) with PDGF-BB. The next day, the medium was removed, the NanoBRET™ NanoLuc Substrate was added, and bioluminescence resonance energy transfer (BRET) was analyzed on a GLOMAX Discover multiplate reader (Promega) at 37 °C using the 450BP (donor) and 600LP (acceptor) built-in filters.

### NIH3T3 foci formation assay

NIH3T3 cells (40,000 cells/well) were seeded the day before into collagen-coated 12-well plates and transfected with 500 ng of DNA (wild-type or mutant pEF-myc-cyto-PDGFRA) and 1.5 µl of Lipofectamine 2000 in 100 µl of Opti-MEM (both from Invitrogen) as described^20^. Twenty-four hours after transfection, three-fifth of the cells from each well were transferred to collagen-coated six-well plates and kept in DMEM with 10% fetal bovine serum and 0.5 mg/ml of G418 until cells reached confluence. Thereafter, cells were kept in DMEM with 5% fetal calf serum and 0.25 mg/ml of G418. Two weeks after transfection, the cells were fixed in methanol and stained with 0.2% crystal violet in 20% ethanol. Foci density was quantified using the Bio1D software (Vilber).

### CellTiter-Glo luminescent cell viability assay

Ba/F3 cells stably expressing wild-type or mutant PDGFRα were washed three times with PBS to remove IL-3. Cells were seeded in 96-well white plates (30,000 cells/well) in complete medium (200 µl) in the presence of PDGF-BB, IL-3, imatinib, dasatinib and/or avapritinib (six replicates per condition) at the indicated concentrations. After 48 h, CellTiter-Glo reagent (100 µl, #G7572, Promega) was added to each well and incubated for 15 min at room temperature. The luminescent signal was measured using a GLOMAX instrument (Promega).

### Statistics

The graphics were generated using Prism software. All experiments were performed at least three times (unless otherwise stated) and produced similar results. The average of different experiments is shown with standard error of the mean (SEM). Statistical analyzes were performed using Student's t-test (*p < 0.5; **p < 0.01; ***p < 0.001), comparing mutants to WT untreated conditions (unless otherwise stated).

## Results

### K385I and K385L mutations constitutively activate PDGFRα signaling

**Figure 1:**
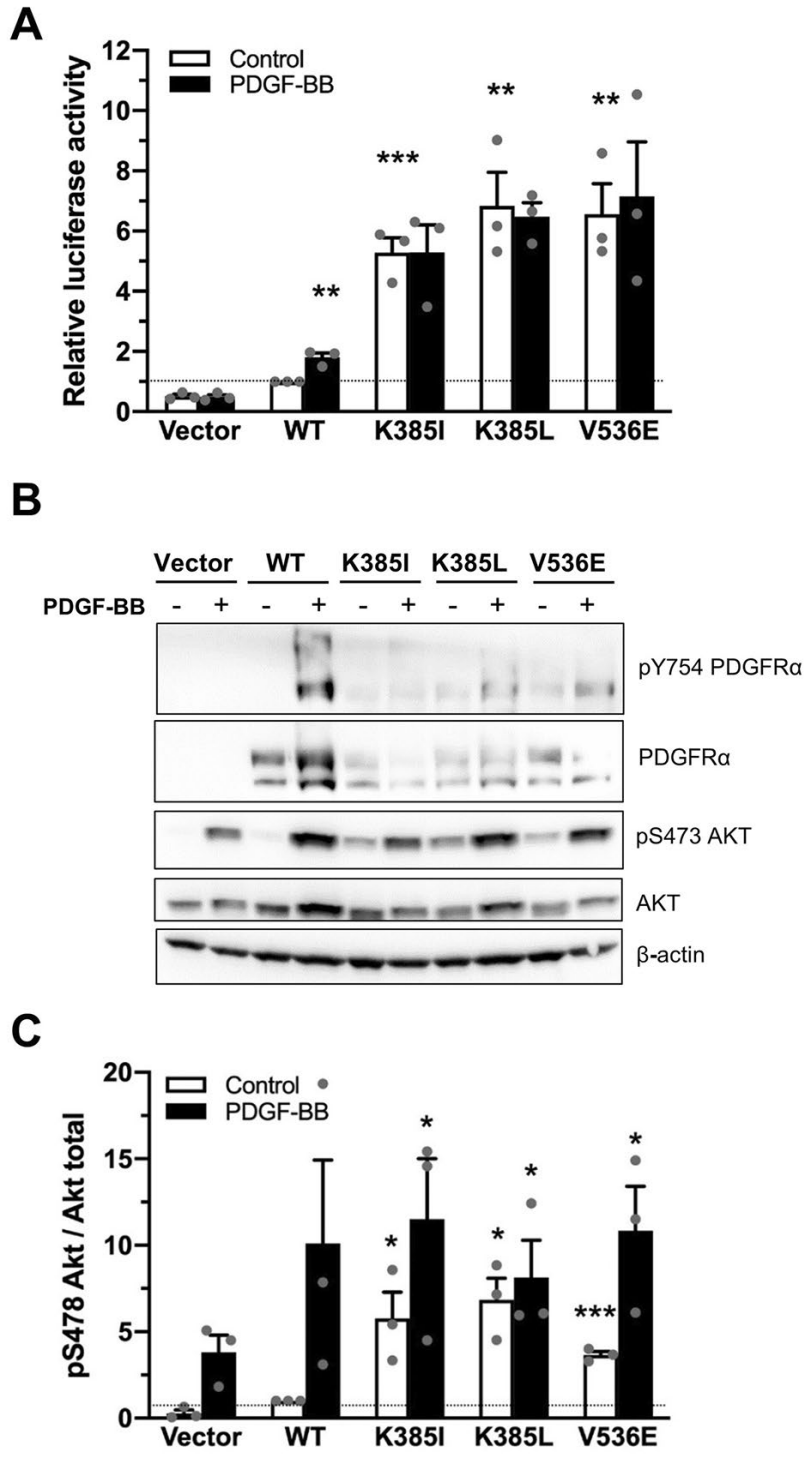
Constitutive signaling by PDGFRα K385I/L receptors. (**A**) U-87 MG glioblastoma cells were co-transfected with the indicated receptor, a luciferase reporter responding to the STAT pathway and a control β-galactosidase reporter. Cells were stimulated or not with PDGF-BB (25 ng/ml) for 24 h. The next day, the luciferase activity was monitored in cell lysates and normalized to the β-galactosidase activity. The histograms represent the mean of three independent experiments with SEM, relative to the WT untreated condition. (**B**) U-87 MG cells were transduced with PDGFRA by retroviral infection. The cells were starved overnight and stimulated for 15 min with PDGF-BB (25 ng/ml) before lysis. Protein extracts were analyzed by western blot with antibodies directed against phosphorylated PDGFRα and AKT. Anti-AKT, anti-PDGFRα and anti-β-actin were used as controls. (**C**) AKT phosphorylation was quantified using Bio1D (Vilber) software and normalized to the WT unstimulated receptor condition. The histograms represent ratios between AKT phosphorylation and AKT expression. The mean of three independent experiments is represented with SEM. Original blots are presented in Supplementary Information.

We first assessed the impact of the K385I/L mutations on the PDGFRα receptor activity. We transfected U-87 MG cells, a human glioblastoma cell line lacking endogenous α receptor expression with a luciferase reporter responsive to STAT transcription factors (STAT1, STAT3, STAT5), a classical readout of PDGF receptor activity^19^. As a positive control, we used the V536E mutation, a known activating mutation identified in glioblastoma^21^ and previously characterized in our laboratory^18^. Our results demonstrated that both K385 mutants exhibited constitutive activity, in a ligand-independent manner (Fig. 1A). In the absence of PDGF-BB, the activity of the mutants was significantly higher than wild-type (WT) PDGFRα.

We next transduced U-87 MG cells with mutant receptors to generate stable cell lines and investigated their downstream signaling by analyzing the phosphorylation of PDGFRα and AKT by western blot. Even though the K385I and K385L mutants were expressed at a lower level than the WT receptor, they were constitutively phosphorylated on tyrosines and induced a ligand-independent phosphorylation of AKT (Fig. 1B,C), further demonstrating that these mutations constitutively activated the receptor when compared to the non-stimulated WT receptor (Fig. 1B).

### K385I/L mutants drive cell proliferation and transformation

**Figure 2:**
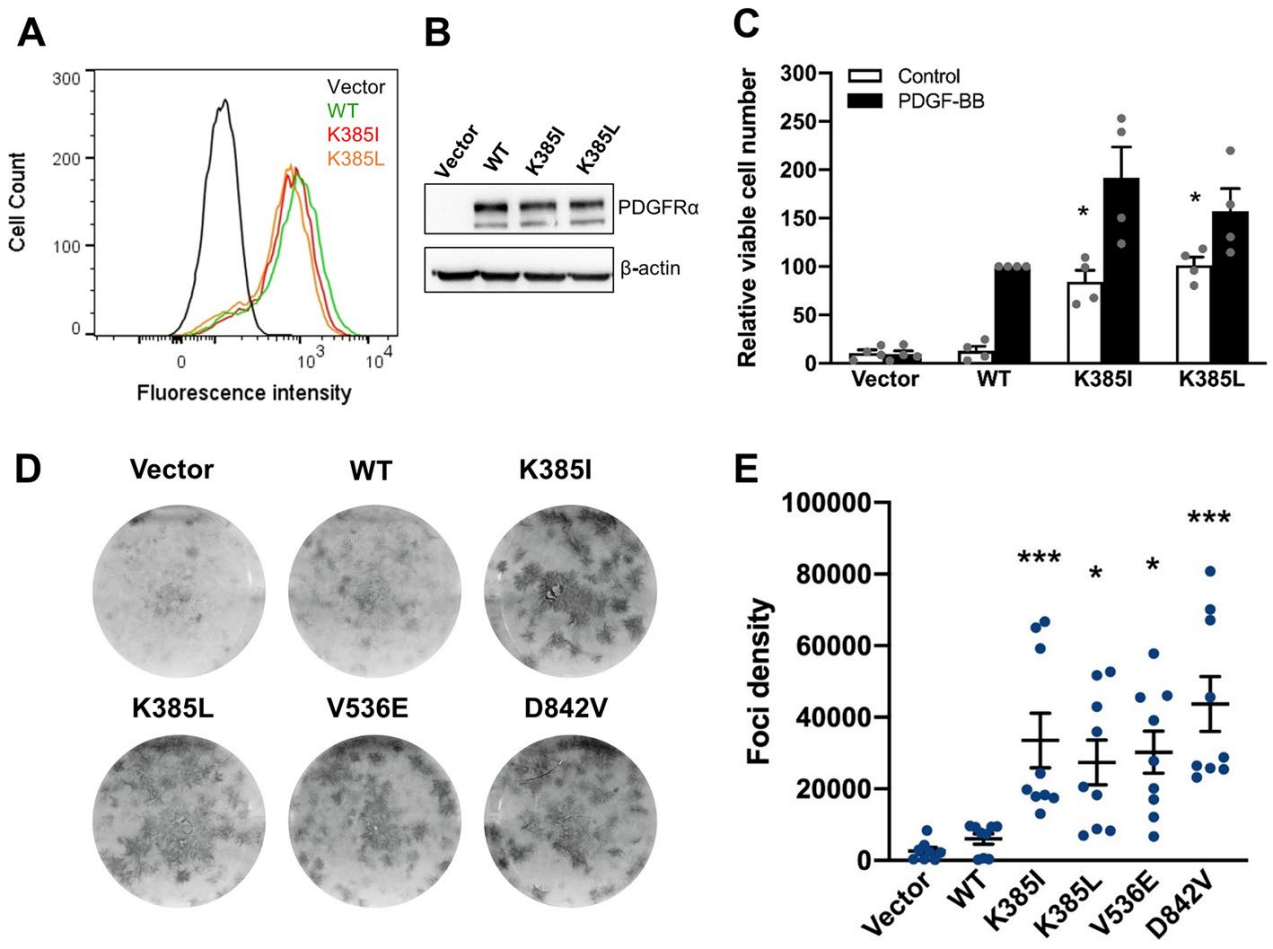
K385I and K385L are oncogenic driver mutations. Ba/F3 cells were transduced with PDGFRA by retroviral infection. Receptor expression was tested by (**A**) flow cytometry or (**B**) by western blot. (**C**) Ba/F3 cell viability and proliferation after IL-3 withdrawal was assessed using CellTiter-Glo luminescent assay. Cells treated or not with PDGF-BB (50 ng/ml) 48 h after removing IL-3. The histograms represent the mean of three independent experiments with SEM, normalized to the WT condition treated with PDGF-BB. (**D**) NIH3T3 cells were transfected with WT or mutated PDGFRα and treated with G418. After approximately 2 weeks, cells were fixed in methanol and colored with crystal violet. (**E**) Foci density was quantified using Bio1D software and normalized to the WT receptor condition. The mean of three independent experiments is represented with SEM (Wilcoxon test; *p < 0.5; ***p < 0.001 compared to WT).

We next evaluated the impact of the mutant receptors on cell proliferation in stably transfected Ba/F3 cells, a well-established model to test the activity of oncogenes^18^. All mutants were expressed at the same level, as shown by western blot, and were detected at the cell surface by flow cytometry (Fig. 2A,B). The parental Ba/F3 cell line requires the presence of interleukin-3 (IL-3) in the culture medium. While Ba/F3 cells expressing PDGFRα WT died shortly after the withdrawal of IL-3, the expression of K385I/L receptors allowed cells to proliferate indefinitely in the absence of IL-3. This was illustrated by a CellTiter Glo assay, which monitors metabolically-active cells by quantifying their ATP content (Fig. 2C). All cell lines proliferated similarly in the presence of IL-3, used as control (data not shown).

To further demonstrate that K385I and K385L are oncogenic mutations, we performed a fibroblast foci formation assay, as described^20,22^. NIH3T3 cells were transfected with K385I, K385L or a positive control, V536E or D842V^23^. In line with the results obtained in Ba/F3 experiments, PDGFRα K385I/L induced the formation of foci, whereas the WT receptor did not (Fig. 2D,E). Altogether, our data demonstrated that K385I/L are oncogenic driver mutations.

### K385I/L mutations promote the receptor dimerization

**Figure 3:**
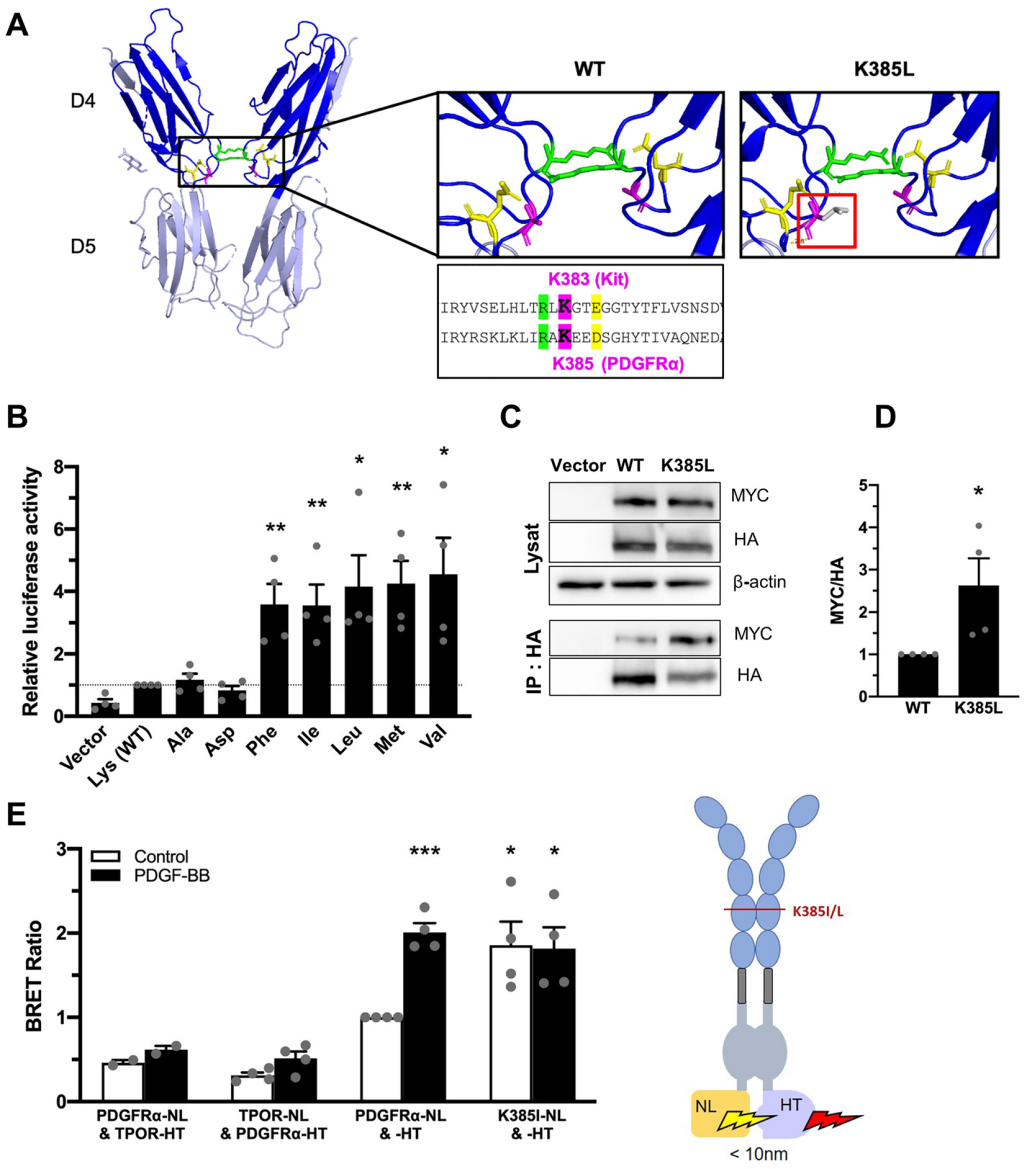
Lysine385 controls PDGFRα receptor activation and dimerization. (**A**) Crystallographic structure of the D4-D5 domain of the KIT receptor (PDB ID: 2E9W, visualized using PyMOL). K381 and E386 are depicted in green and yellow, respectively. On the left panel (WT), the lysine at position 383 in KIT is shown in pink. On the right panel (K385L), the lysine at position 383 in KIT was replaced by a leucine (in gray in the red frame). (**B**) U-87 MG cells were co-transfected with the indicated codon 385 mutant, a luciferase reporter responding to the STAT pathway and a control β-galactosidase reporter. After 24 h, the luciferase activity was monitored in cell lysates and normalized to the β-galactosidase activity. The histograms represent the mean of four independent experiments with SEM, normalized on WT. (**C**) HEK293T cells were co-transfected with HA- and MYC-tagged PDGFRα, WT or mutated. Proteins were extracted and immunoprecipitated with an anti-HA antibody. Then, a western blot was performed with an anti-MYC antibody. (**D**) Anti-Myc and -HA western blots were quantified using Bio1D software and normalized to the WT receptor. The histograms represent ratios between Myc and HA signals. The mean of four independent experiments is represented with SEM. (**E**) NanoBRET between WT or mutant PDGFRα coupled to nanoluciferase (NL) and HaloTag (HT). The unstructured C-terminal tail (110 residues) was removed in all constructs. TPOR-NanoLuciferase (TPOR-NL) and TPOR-HaloTag (TPOR-HT) were used as controls. The mean of four independent experiments is represented with SEM.

K385I/L mutations are located within the immunoglobulin-like D4 domain, which is known to be involved in receptor dimerization^24^. Since the crystallographic structure of the extracellular region of PDGFRα has not been characterized yet, we relied on the structure of the highly related KIT receptor^25^. Figure 3A illustrates the molecular structure of the extracellular D4 and D5 domains of KIT. The lysine at position 385 is conserved among receptors of the same family, and is located between an arginine and a glutamate that are essential for KIT and PDGFRβ activation^16^. The substitution of K385 by a hydrophobic amino acid such as leucine or isoleucine could create a hydrophobic bond between two D4 domains and therefore enhance dimerization, as suggested by Chen and colleagues^26^.

To explore this hypothesis, we mutated lysine 385 into other amino acids including hydrophobic residues (alanine, aspartate, phenylalanine, methionine, valine). The mutants K385A and K385D did not activate the receptor as their activity was similar to PDGFRα WT (Fig. 3B), suggesting that the removal of the lysine is not enough to activate the receptor. By contrast, the variants K385F, K385M, and K385V behaved similarly to the K385I/L mutants (Fig. 3B). These results suggest that the addition of another hydrophobic amino acid at position K385 constitutively activated the receptor, despite the variable size of the side chains of valine, methionine, and phenylalanine (Fig. 3B).

We next tested whether K385 mutations affected the receptor dimerization. We first transfected cells with both HA- and MYC-tagged monomeric receptors and conducted a co-immunoprecipitation experiment followed by western blot. In the absence of ligand, we observed a weak co-precipitation of tagged WT receptors (Fig. 3C). The K385L mutation significantly stabilized the interaction between HA- and MYC-tagged receptors. Quantification of the bands suggested that the mutant increased dimerization approximately threefold compared to the WT receptor.

To further test for constitutive dimerization of PDGFRα receptors in living cells, we took advantage of bioluminescence resonance energy transfer (BRET) between nanoluciferase (NL) and HaloTag (HT), fused to the C-terminus of PDGFRα. We removed the unstructured C-terminus of the receptor (the last 110 residues), to limit the analysis to short distance interactions (< 10 nm). A strong BRET signal was detected from the mutant receptors (K385I-NL &-HT) as well as the WT receptors stimulated with PDGF-BB (PDGFRα-NL &-HT) (Fig. 3E). As controls, we used thrombopoietin receptors (TPOR) fused to nanoluciferase and HaloTag, which were previously described and did not interact with the corresponding PDGFRα constructs^27^.

Altogether, these results suggested that the addition of a hydrophobic residue at position 385 increases PDGFRα dimerization, which could be responsible to the oncogenic activity of these mutants.

### PDGFRα K385I/L mutants are sensitive to tyrosine kinases inhibitors

**Figure 4:**
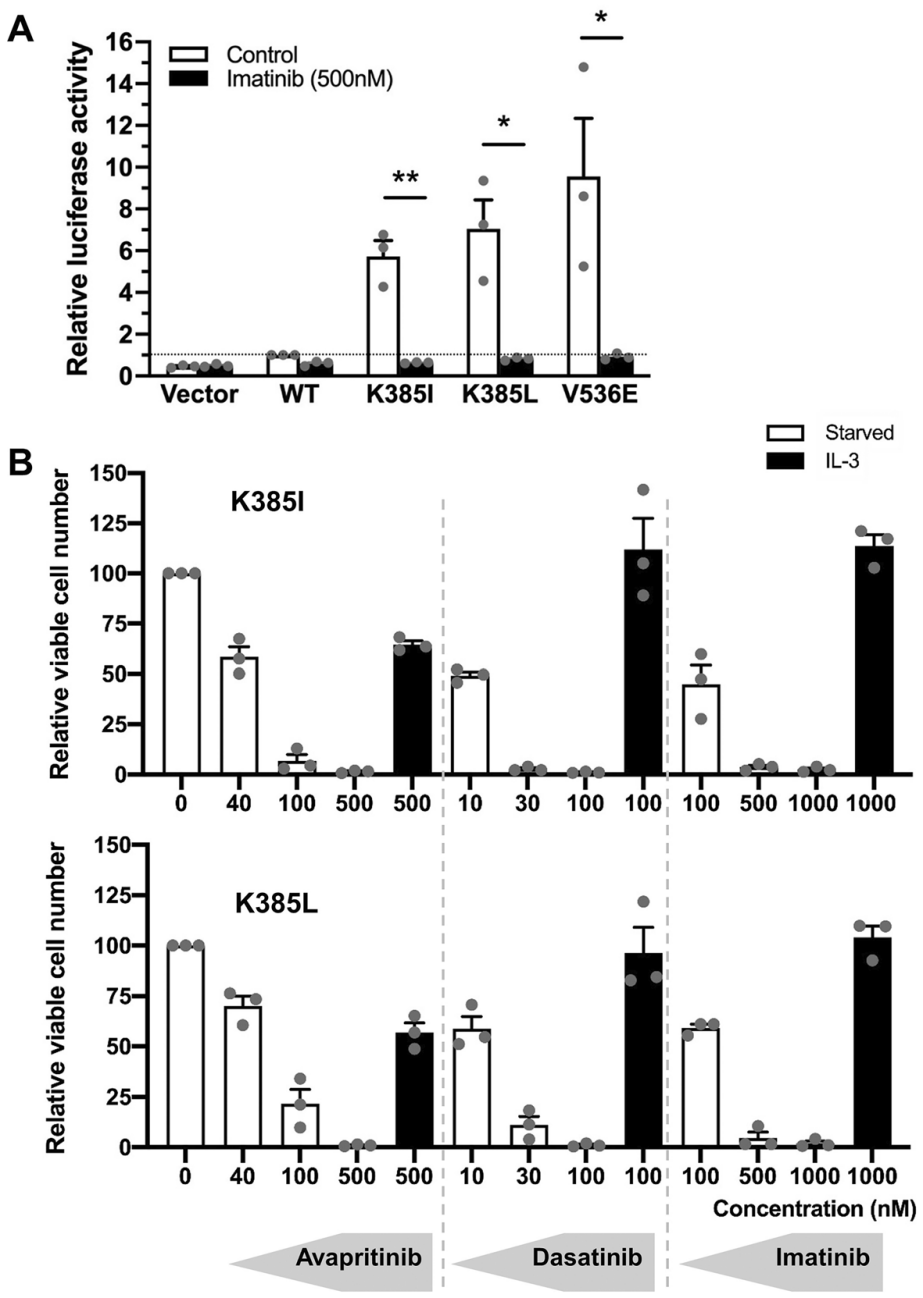
PDGFRα K385I/L receptors are sensitive to imatinib, dasatinib and avapritinib. (**A**) U-87 MG cells were co-transfected with the indicated receptor and a luciferase reporter as described above. The histograms represent the mean of three independent experiments with SEM, normalized to the WT untreated condition. (**B**) A CellTiter-Glo luminescent assay was conducted on Ba/F3 cells expressing WT or mutated PDGFRα treated with increasing concentrations of imatinib, dasatinib, or avapritinib for 24 h. The histograms represent the mean of three independent experiments with SEM, normalized to the untreated condition.

Some PDGFRα mutants (such as V536E) can be blocked by the first generation TKI imatinib, while others (like D842V) are resistant to this drug^18^. Interestingly, luciferase reporter assays revealed that K385I/L mutants were sensitive to imatinib at a clinically relevant concentration (500 nM) (Fig. 4A).

We further tested the sensitivity of mutants in stable Ba/F3 cell lines. In addition to imatinib, we evaluated the second and third generation inhibitors dasatinib and avapritinib (Fig. 4B). Ba/F3 cells that relied on the expression of PDGFRα K385I/L to survive in IL-3-free medium died when treated with imatinib, dasatinib and avapritinib (Fig. 4B). This effect was reversed by adding exogenous IL-3 to the culture medium, demonstrating the specificity of the inhibition.

## Discussion

Our results demonstrate that PDGFRα K385I and K385L constitutively activate the receptor through dimerization. Luciferase assays, western blot, and cell transformation assays, highlighted the oncogenic potential of the K385I/L mutations. Our study provides the first experimental characterization of these unique mutations.

Co-immunoprecipitation experiments and BRET assays revealed that K385 mutants showed a significantly higher level of dimerization. Collectively, these results strongly support the hypothesis of ligand-independent constitutive dimerization as the primary mechanism of activation for these mutants. It is worth noting that our results do not rule out the formation of higher order receptor multimers. It is also possible that dimerization alone is not sufficient to explain the constitutive activity of the receptor, and other factors may come into play. Indeed, PDGFRα Lys385 (equivalent to Lys387 in PDGFRβ and Lys383 in the KIT receptor, depicted in pink in Fig. 3) is located between two amino acids previously described as important for receptor activation^16^. Arg385 and Glu390 in PDGFRβ (equivalent to Arg383 and Asp388 in PDGFRα and Arg381 and Glu386 in KIT, depicted in green and yellow, respectively, in Fig. 3) form two pairs of salt bridges, which are necessary for receptor activation upon ligand binding. According to Yang and colleagues, these interactions allow for precise orientation of the receptor to the plasma membrane, facilitating the activation of the cytoplasmic domains of the two receptors. Changing these two residues to alanine did not affect dimerization but resulted in a loss of receptor activity, suggesting that dimerization alone is not sufficient to activate the receptor^16^. Hence, the K385I/L mutations may stabilize the active conformation of the dimeric receptor in addition to promoting dimerization. In agreement with this hypothesis, based on an in silico structural analysis, Chen and colleagues had suggested previously that a methionine at position 385 may promote a small hydrophobic patch adjacent to several salt-bridges, enhancing receptor dimerization and activation^26^. To our knowledge, this work provides the first experimental evidence for enforced PDGFRα dimerization by a point mutation. Although dimerization is the natural mechanism of PDGF receptor activation by their ligands, most PDGF receptor alterations affect the receptor intracellular part, resulting in the direct activation of the tyrosine kinase domain. Nevertheless, oligomerization is the driving force of *PDGFRB* fusion genes, such as *ETV6-PDGFRB*^28^.

*PDGFRA* somatic alterations in the extracellular domain are restricted to some cancer types, such as gliomas, and their contribution to tumor development remains unclear^18,29^. Recently, overexpression and trapping in the endoplasmic reticulum was identified as a novel mechanism of *PDGFRA* activation by an extracellular Y288C mutation in the D3 domain^29^. Interestingly, other somatic alterations of the D4 domain sequence were reported in brain tumors unrelated to MGNT. In adult patients, glioma tumors with a *PDGFRA* amplification frequently harbor a genomic deletion of exons 8 and 9, which encode part of D4 and D5^30^. This deletion has been identified as oncogenic, but the precise mechanism of receptor activation remains elusive.

*PDGFRA* K385I/L mutations are restricted to MGNT. The Catalog of Somatic Mutations In Cancer (COSMIC) reports a single exception: a glioma case with a K385I mutation^31^. Four cases of astrocytoma with a K385M substitution are also present in COSMIC. This mutation requires a single nucleotide change (AAG > ATG) and activated the receptor as efficiently as K385I/L in our luciferase assay. However, it has not been found in MGNT.

Two models could explain the unique association of K385I/L mutations with MGNT. First, these mutations could provide a selective advantage when occurring in the cell of origin of MGNT. Future work will have to investigate whether K385I/L mutations have a cell type specific effect. However, this model does not explain why dinucleotide changes at the K385 codon are present in MGNT, instead of the more likely single nucleotide mutation required for generating the K385M mutant. Our data also shows that the mutants behave as oncogenes in classical assays based on Ba/F3 and NIH3T3 cells. The alternative model posits the existence of a unique mutagenesis process responsible for this unusual dinucleotide substitution in the cells of origin of these tumors. Further studies may unravel a specific mechanism of DNA damage and repair.

Our study offers valuable insights into potential therapeutic implications for patients with MGNT. We tested three tyrosine kinase inhibitors, imatinib, dasatinib, and avapritinib and observed their ability to effectively inhibit the constitutive activity of mutant receptors. Imatinib is an FDA-approved molecule since 2001 and is widely used for the treatment of various cancers, particularly in chronic myeloid leukemia characterized by the presence of the BCR-ABL fusion gene. However, imatinib shows a limited penetration across the blood–brain barrier^32,33^. Therefore, we investigated tyrosine kinase inhibitors that effectively cross the blood–brain barrier, such as dasatinib and avapritinib. On the one hand, preclinical studies have revealed that dasatinib improves the survival of patients with Philadelphia chromosome-positive acute intracranial leukemia, particularly when imatinib failed to inhibit tumor growth^34^. On the other hand, avapritinib has shown durable clinical efficacy in the treatment of gastrointestinal stromal tumors (GIST) with activating mutations in KIT and PDGFRα, which were previously considered untreatable due to resistance to TKI^35^. Additionally, avapritinib demonstrated encouraging results in a patient who developed central nervous system metastasis^36^. Nevertheless, this drug also showed increased neurological side effects. Altogether, our results suggested that dasatinib and avapritinib are two tyrosine kinase inhibitors which may have therapeutic potential for treating patients with MGNT tumors.

A limitation of the present study lies in the lack of appropriate MGNT preclinical models to test the effects of mutations and inhibitors. In the absence of MGNT primary cell culture, cell line or animal model, we used cell lines that have been validated to test the activity of classical oncogenes. These cellular models demonstrated the oncogenic activity of the mutants and their sensitivity to TKI, but may not recapitulate all molecular aspects of MGNT. In the future, the development of more relevant MGNT preclinical models will be critical to validate new treatments.

In conclusion, our study provides novel insights into the unique features of the K385I/L mutations specific to myxoid glioneuronal tumors and their molecular mechanism, involving ligand-independent increased dimerization. These findings establish a basis for potential targeted therapeutic approaches based on tyrosine kinase inhibitors in the treatment of MGNT patients, offering hope for improved clinical outcomes in this challenging disease.

### Supplementary Information

Supplementary Figures.

Supplementary Tables.

## Data Availability

The data that support the findings of this study are available from the corresponding author upon reasonable request.
