# Supplementary material for: PDGFRA K385 mutants in myxoid glioneuronal tumors promote receptor dimerization and oncogenic signaling
Source: Sci Rep. 2024 Mar 26;14:7204. doi: 10.1038/s41598-024-57859-5 (PMC10965988; doi:10.1038/s41598-024-57859-5)

PDGFRA K385 mutants in myxoid glioneuronal tumors promote receptor dimerization and oncogenic signaling
Laurence de Villenfagne, Ariane Sablon, Jean-Baptiste Demoulin

Supplementary data for figure 1B : uncropped image

N1

Raw image generated by FUSION Solo S (Vilber) | Image modified to reveal edges

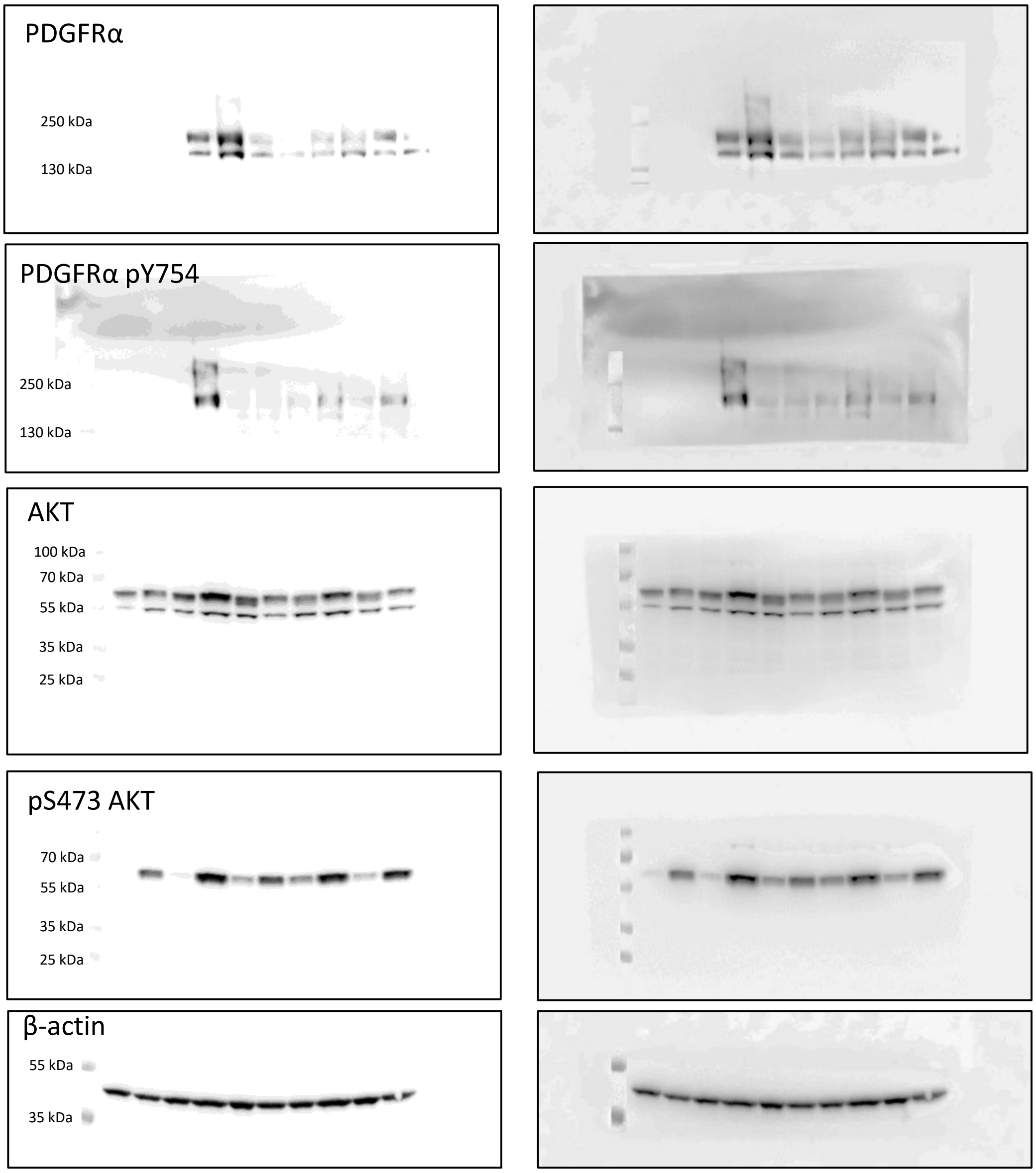

Supplementary data for figure 1B : uncropped image

N2

Raw image generated by FUSION Solo S (Vilber) | Image modified to reveal edges

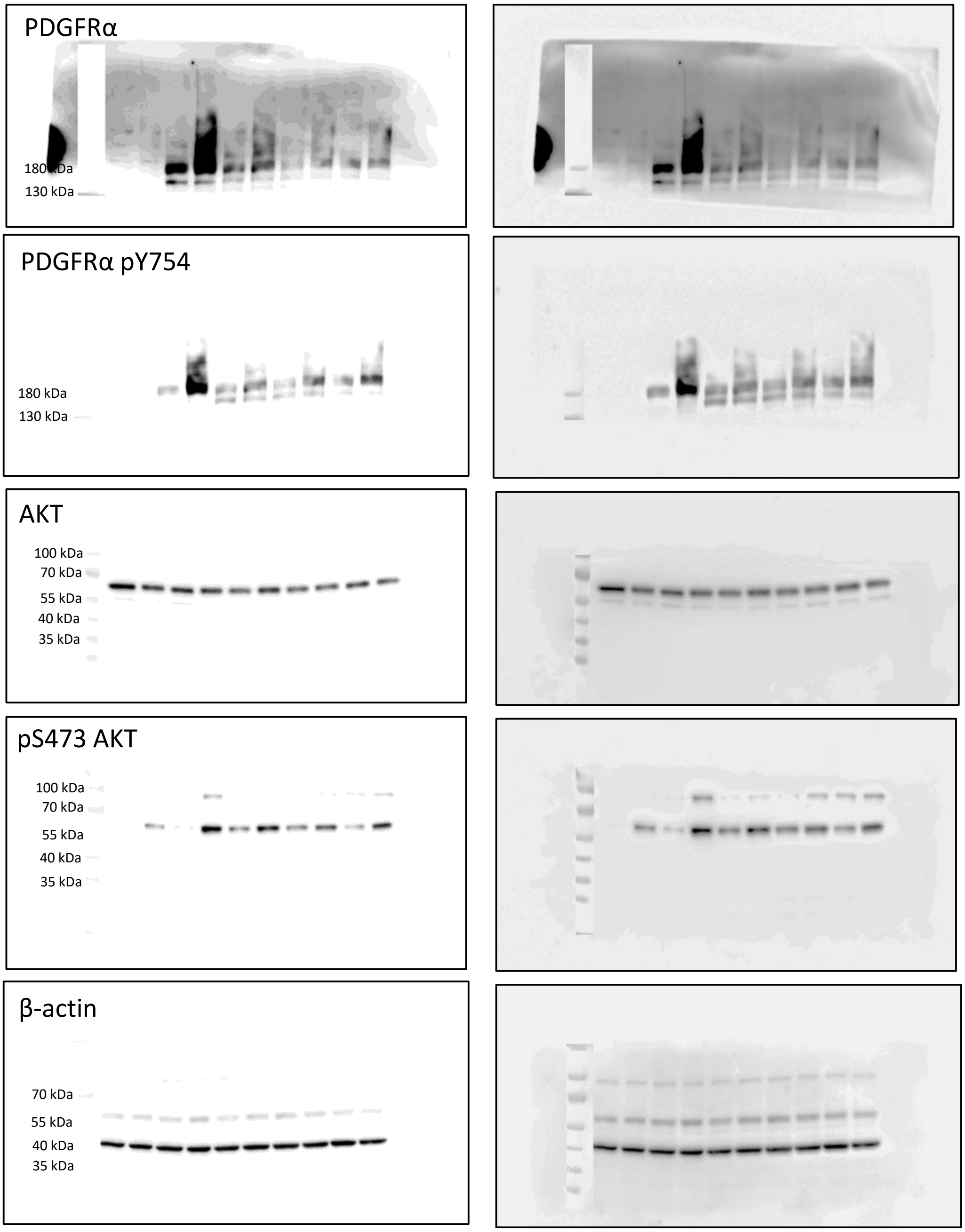

Supplementary data for figure 1B : uncropped image

N3

Raw image generated by FUSION Solo S (Vilber)

Image modified to reveal edges

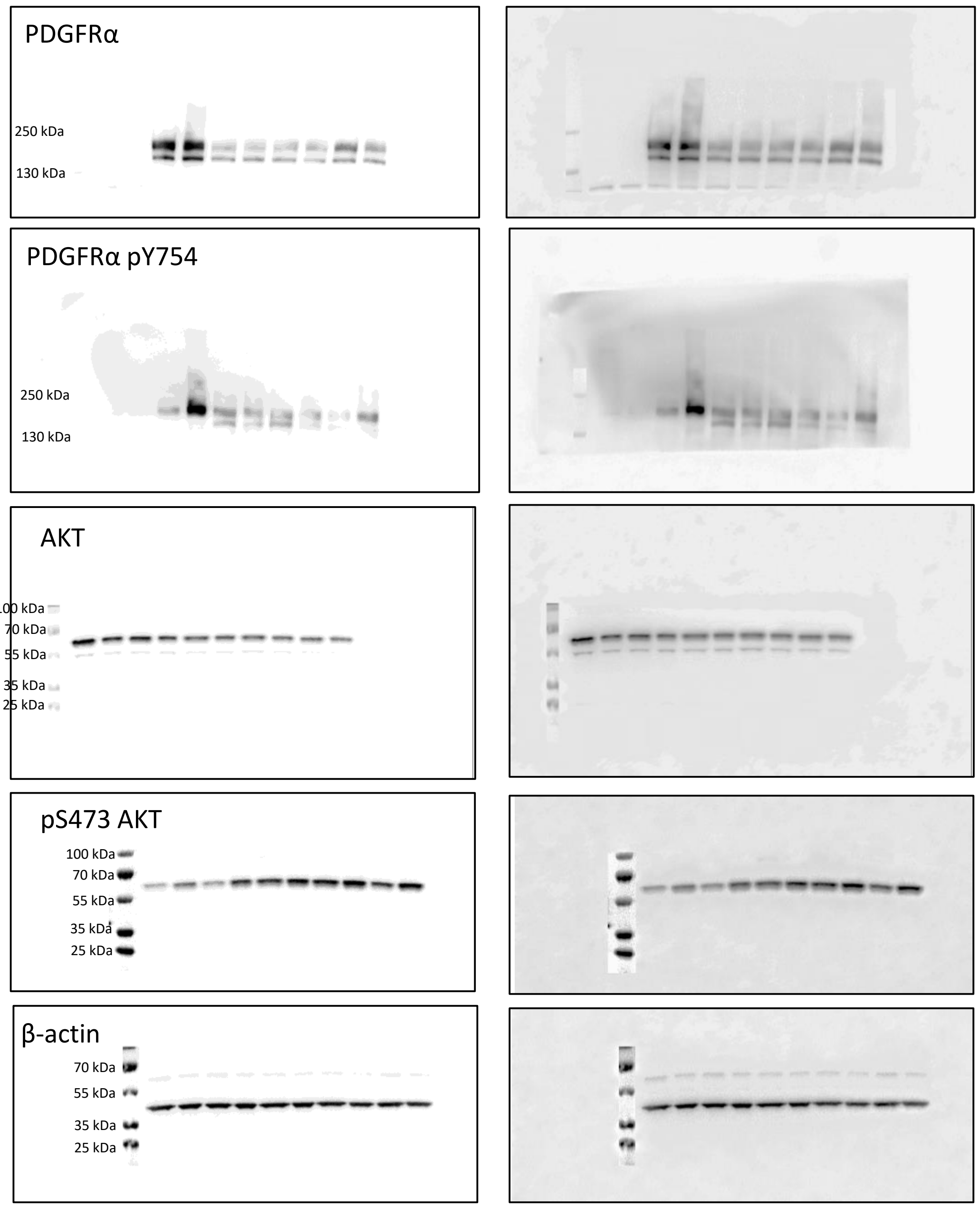

Supplementary data for figure 2B : uncropped image

Raw image generated by FUSION Solo S (Vilber)

Image modified to reveal edges

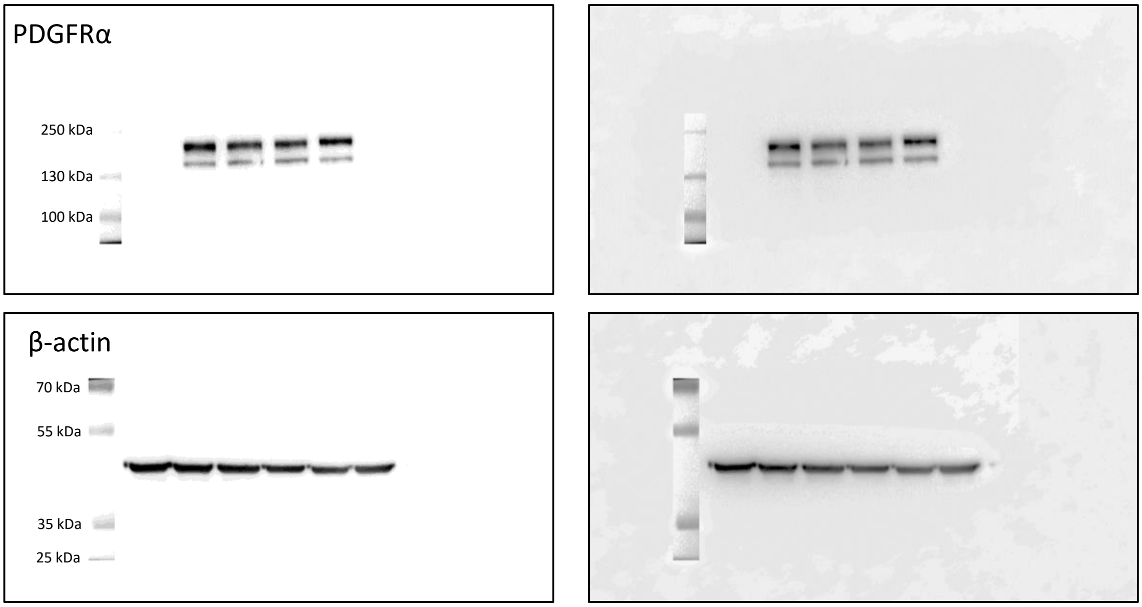

Supplementary data for figure 3C : uncropped image

N1

Raw image generated by FUSION Solo S (Vilber) | Image modified to reveal edges

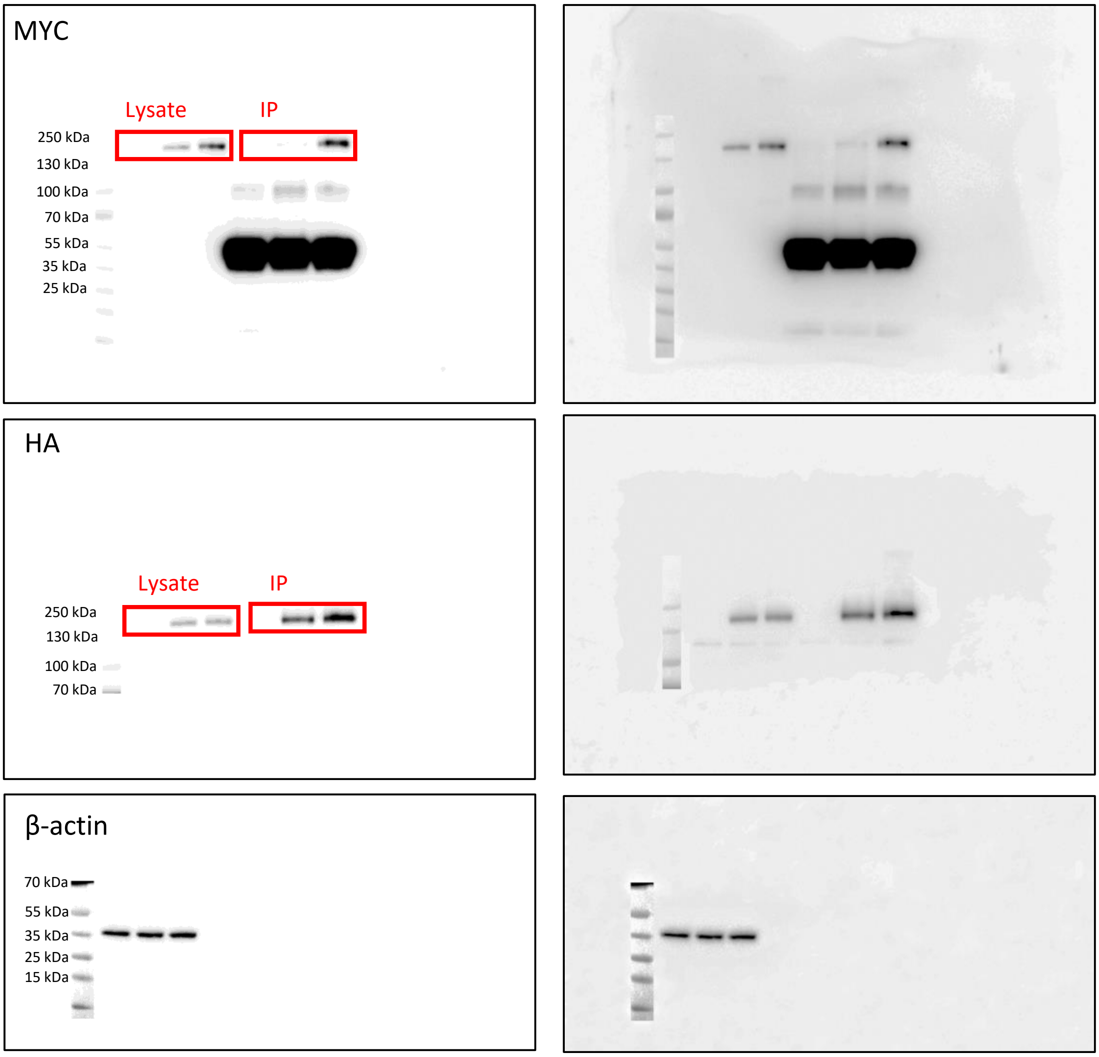

Supplementary data for figure 3C : uncropped image

N2

Raw image generated by FUSION Solo S (Vilber)

Image modified to reveal edges

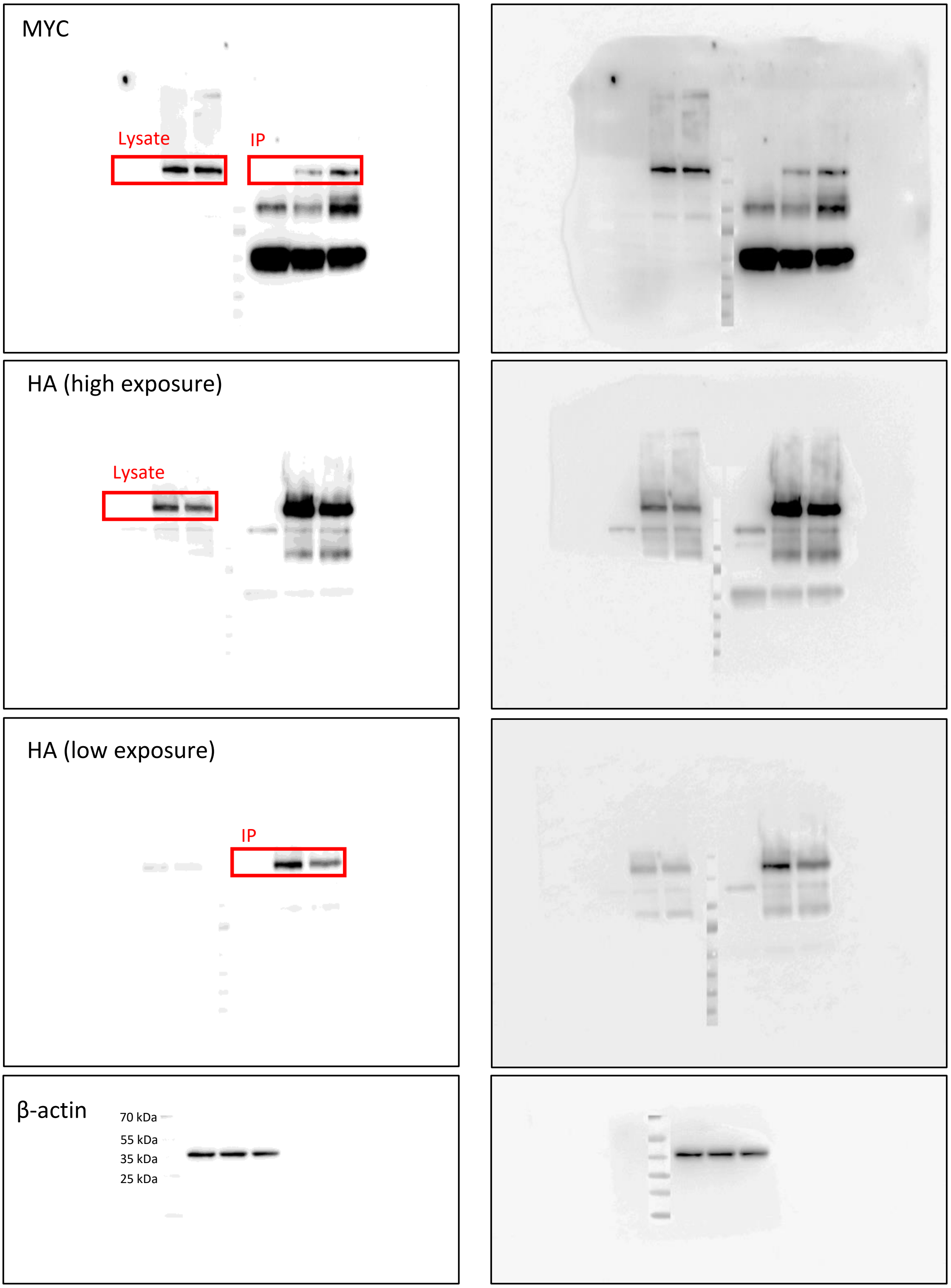

Supplementary data for figure 3C : uncropped image

N3

Raw image generated by FUSION Solo S (Vilber) | Image modified to reveal edges

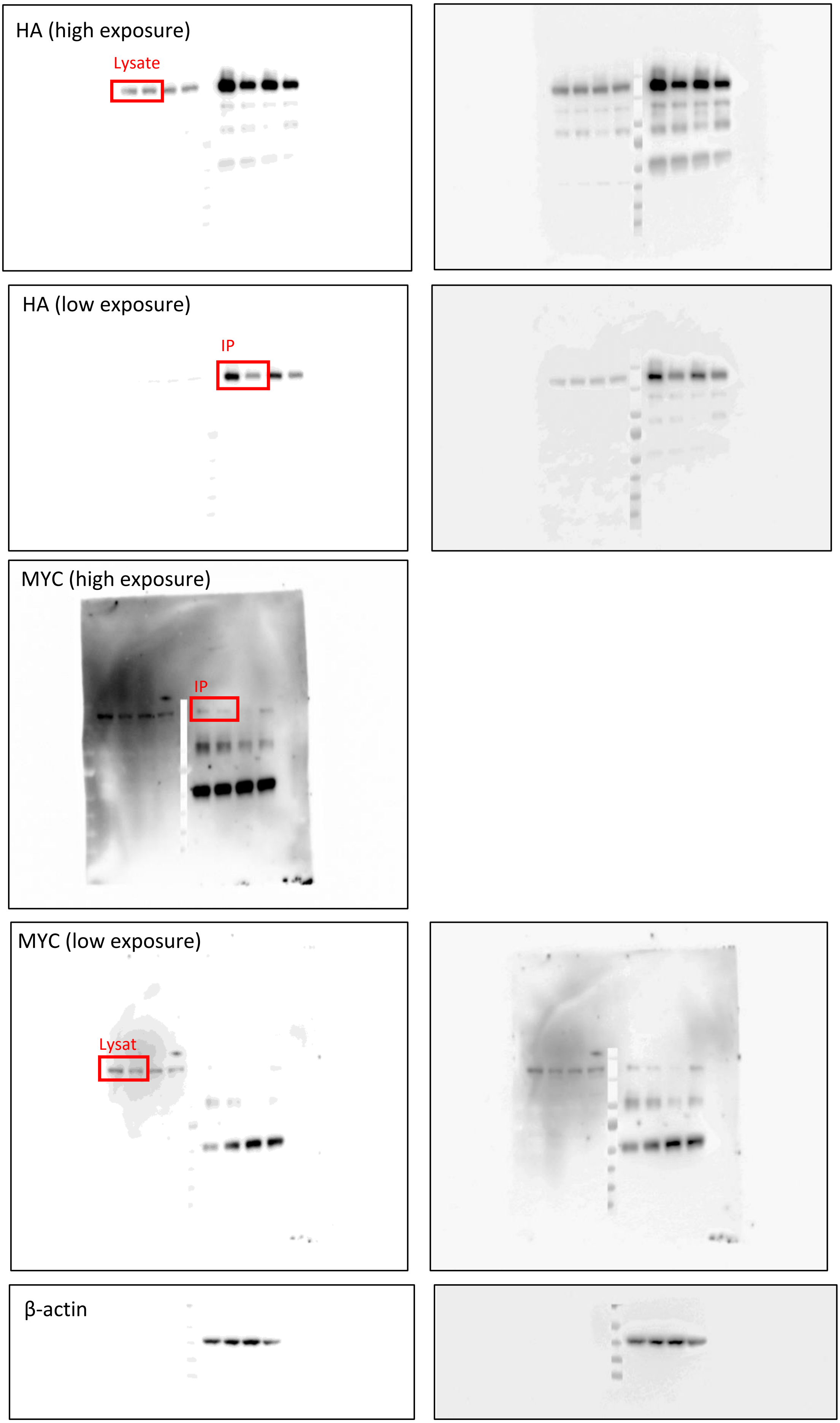

Supplement: Supplementary file 1 — Supplementary Figures. [file 41598_2024_57859_MOESM1_ESM.pdf]
