# Supplementary material for: PDGFRA K385 mutants in myxoid glioneuronal tumors promote receptor dimerization and oncogenic signaling
Source: Sci Rep. 2024 Mar 26;14:7204. doi: 10.1038/s41598-024-57859-5 (PMC10965988; doi:10.1038/s41598-024-57859-5)
Supplement: Supplementary file 2 — Supplementary Tables. [file 41598_2024_57859_MOESM2_ESM.pdf]

PDGFRA K385 mutants in myxoid glioneuronal tumors promote receptor dimerization and oncogenic signaling
Laurence de Villenfagne, Ariane Sablon, Jean-Baptiste Demoulin

Supplementary information: sequence of the different oligonucleotides used

K385A	Forward<br>Reverse	aaattaaagctgatccgtgctgcggaagaagacagtggcca<br>tggccactgtcttcttccgcagcacggatcagctttaattt
K385D	Forward<br>Reverse	caaattaaagctgatccgtgctgatgaagaagacagtggccattata<br>tataatggccactgtcttcttcatcagcacggatcagctttaatttg
K385F	Forward<br>Reverse	gcaaattaaagctgatccgtgctttcgaagaagacagtggccattatac<br>gtataatggccactgtcttcttcgaaagcacggatcagctttaatttgc
K385I	Forward<br>Reverse	caaattaaagctgatccgtgctatagaagaagacagtggccattatac<br>gtataatggccactgtcttcttctatagcacggatcagctttaatttg
K385L	Forward<br>Reverse	aaattaaagctgatccgtgctttggaagaagacagtggcca<br>tggccactgtcttcttccaaagcacggatcagctttaattt
K385M	Forward<br>Reverse	taaagctgatccgtgctatggaagaagacagtgg<br>ccactgtcttcttccatagcacggatcagcttta
K385V	Forward<br>Reverse	aaattaaagctgatccgtgctgtggaagaagacagtggcca<br>tggccactgtcttcttccacagcacggatcagctttaattt
V536E	Forward<br>Reverse	gtcctggtgctgttggagattgtgatcatctc<br>gagatgatcacaatctccaacagcaccaggac
D842V	Forward<br>Reverse	ctttggcctggccagagtcatcatgcatgattcga<br>tcgaatcatgcatgatgactctggccaggccaaag
Cloning PDGFRα into a Nano-Luciferase vector	Forward<br>Reverse<br>Forward<br>Reverse	ggctcgagcggcgtcttc<br>ggcggcgaattcggaagc<br>tccgaattcgccgccatggggacttcccatccgg<br>gacgccgctcgagcccaggaagctgtcttccaccagg
Cloning PDGFRα into a Halotag vecor	Forward<br>Reverse<br>Forward<br>Reverse	ccgatgcagctgccttatgac<br>gggcgaattcggaagcgatc<br>cttccgaattcgcccatggggacttcccatccgg<br>aggcagctgcatcggcaggaagctgtcttccaccagg
